# Risk factors for recurrent cerebral amyloid angiopathy-related intracerebral hemorrhage

**DOI:** 10.3389/fneur.2023.1265693

**Published:** 2023-11-07

**Authors:** Xinglei Jia, Menghan Bo, Hong Zhao, Jia Xu, Luqian Pan, Zhengyu Lu

**Affiliations:** ^1^VIP Department, Longhua Hospital, Shanghai University of Traditional Chinese Medicine, Shanghai, China; ^2^Teaching Affairs Department, Yueyang Hospital of Integrated Traditional Chinese and Western Medicine, Shanghai University of Traditional Chinese Medicine, Shanghai, China; ^3^Department of Geriatrics, Longhua Hospital, Shanghai University of Traditional Chinese Medicine, Shanghai, China

**Keywords:** intracerebral hemorrhage, cerebral amyloid angiopathy, cerebral small vessel disease, cortical superficial siderosis, recurrence

## Abstract

**Background:**

Cerebral amyloid angiopathy (CAA) is the most common cause of lobar intracerebral hemorrhage (ICH) in the elderly, and its multifocal and recurrent nature leads to high rates of disability and mortality. Therefore, this study aimed to summarize the evidence regarding the recurrence rate and risk factors for CAA-related ICH (CAA-ICH).

**Methods:**

We performed a systematic literature search of all English studies published in PubMed, Embase, Web of Science, Cochrane Library, Scopus, and CINAHL from inception to June 10, 2023. Studies reporting CAA-ICH recurrence rates and risk factors for CAA-ICH recurrence were included. We calculated pooled odds ratios (ORs) with their corresponding 95% confidence intervals (CIs) using a random/fixed-effects model based on the I^2^ assessment of heterogeneity between studies. Publication bias was assessed using Egger's test.

**Results:**

Thirty studies were included in the final analysis. Meta-analysis showed that the recurrence rate of CAA-ICH was 23% (95% CI: 18–28%, I^2^ = 96.7%). The risk factors significantly associated with CAA-ICH recurrence were: previous ICH (OR = 2.03; 95% CI: 1.50–2.75; I^2^ = 36.8%; *N* = 8), baseline ICH volume (OR = 1.01; 95% CI: 1–1.02; I^2^ = 0%; *N* = 4), subarachnoid hemorrhage (cSAH) (OR = 3.05; 95% CI: 1.86–4.99; I^2^ = 0%; *N* = 3), the presence of cortical superficial siderosis (cSS) (OR = 2.04; 95% CI: 1.46–2.83; I^2^ = 0%; *N* = 5), disseminated cSS (OR = 3.21; 95% CI: 2.25–4.58; I^2^ = 16.0%; *N* = 6), and centrum semiovale-perivascular spaces (CSO-PVS) severity (OR = 1.67; 95% CI: 1.14–2.45; I^2^ = 0%; *N* = 4).

**Conclusion:**

CAA-ICH has a high recurrence rate. cSAH, cSS (especially if disseminated), and CSO-PVS were significant markers for recurrent CAA-ICH. The onset of ICH in patients with CAA is usually repeated several times, and recurrence is partly related to the index ICH volume. Identifying clinical and neuroimaging predictors of CAA-ICH recurrence is of great significance for evaluating outcomes and improving the prognosis of patients with CAA-ICH.

**Systematic review registration:**

https://www.crd.york.ac.uk/PROSPERO/display_record.php?RecordID=400240, identifier [CRD42023400240].

## 1. Introduction

Cerebral amyloid angiopathy (CAA) is an age-related cerebral small vessel disease (SVD) characterized by the progressive deposition of amyloid-β in the vessel walls of the middle and small arteries and capillaries of the cerebral cortex, covering the soft meninges (1, 2). Among the various phenotypes of CAA, spontaneous intracerebral hemorrhage (ICH) is one of the most widely studied, accounting for approximately 5–20% of spontaneous ICH in the elderly (3). Spontaneous ICH is a worldwide public health problem with a mortality rate of 50% within 30 days, and only 20% of patients regain functional independence at 6 months (4). Unlike hypertensive ICH, which usually presents as a deep hemorrhage (usually in the basal ganglia, thalamus, and brainstem), cerebral amyloid angiopathy-related ICH (CAA-ICH) preferentially affects the cortical or cortico-subcortical (lobar) regions, with multiple and recurrent lobar hemorrhages as its typical features (5). Compared with other types of ICH, CAA-ICH is milder when it first occurs (6); however, the risk of recurrence is significantly higher (7). Therefore, the prognosis is often worse and can lead to a higher risk of dementia, disability, and even death.

Because of the superficial location of CAA-ICH, surgical removal of the intracerebral hematoma is currently an effective and feasible surgical treatment; however, its safety is still controversial, and its suitability for patients with recurrent ICH remains undetermined (8–12). In addition to surgery, specific treatments are currently lacking. Secondary prevention of spontaneous ICH is currently an accepted conservative treatment that includes hemostasis, strict blood pressure control, and brain support therapy (4). Given the absence of targeted treatment, we should focus on the early identification of potential risk factors for the recurrence of CAA-ICH, which is of great significance in preventing the recurrence of ICH and improving disease prognosis.

There may be a significant unrecognized burden of CAA in older populations until now (13). The number of studies on CAA-ICH is limited and there is much room for future exploration. Recent meta-analyses and systematic reviews have shown that neuroimaging markers of SVD are not only related to the pathophysiology of CAA and participate in the diagnosis of CAA, but also play a significant role in predicting the outcome after the occurrence of CAA-ICH (14–16). Computed tomography (CT) and magnetic resonance imaging (MRI) are essential examinations for patients with acute ICH. Neuroimaging markers provide a great opportunity to explore the risk factors associated with CAA-ICH recurrence and predict rebleeding. MRI-based biomarkers of CAA-associated small vessel injury, including cerebral microbleeds (CMBs), cortical superficial siderosis (cSS), centrum semiovale-perivascular spaces (CSO-PVS), and white matter hyperintensities (WMH), have been widely investigated and considered for the diagnosis of CAA and prediction of ICH recurrence (17, 18). These MRI markers may be closely associated with the pathophysiology of CAA-ICH. Therefore, it has been proposed that setting an ordinal scale representing the total burden of small vessel disease in CAA by considering the four most characteristic MRI markers mentioned above seems to have a greater advantage in predicting CAA-ICH recurrence (19). In addition, for patients with acute ICH, CT is easier to perform within a short period; therefore, the baseline CT imaging characteristics of ICH are also worthy of attention. However, the number of studies on the risk factors for CAA-ICH recurrence is limited, and the factors that strongly suggest the recurrence of CAA-ICH are still inconclusive. This has caused great trouble in the treatment and prevention of recurrence in patients with CAA-ICH.

This study aimed to conduct a systematic review and meta-analysis of previous studies to estimate the CAA-ICH recurrence rate and summarize the risk factors associated with its recurrence to improve the prognosis and treatment outcomes of patients at a high risk of recurrence.

## 2. Materials and methods

This systematic review followed the Preferred Reporting Items for Systematic Reviews and Meta-Analyses (PRISMA) guidelines (20) (Supplementary Table 1). The study protocol was registered in the International Registry of Prospective Systematic Reviews (PROSPERO; registration no.: CRD42023400240).

### 2.1. Search strategy

Two authors systematically and independently searched for literature published in PubMed, EMBASE, Web of Science, Cochrane Library, Scopus, and CINAHL from the establishment of the database until June 10, 2023. We combined the subject words “cerebral amyloid angiopathy,” “cerebral hemorrhage,” and related free words to construct a search strategy to obtain relevant literature on the prevalence of recurrence and risk factors for CAA-ICH. The search was not limited to the characteristics of the study population (age, sex, or race). The list of references for each study included in the meta-analysis was manually reviewed to identify other potentially eligible studies. The full search strategy is shown in Supplementary Table 2.

### 2.2. Inclusion and exclusion criteria

The titles and abstracts of all search results were screened first, and the full text of the qualified literature was independently reviewed by two authors. Disagreements were resolved by consensus and consultation with senior investigators.

Studies included in this review had to meet the following criteria: (1) participants were survivors of acute lobar ICH events, regardless of age or sex; (2) the participants were diagnosed with CAA based on histology or Boston diagnostic criteria (In fact, the Boston criteria encompass histologic diagnosis. Histological diagnosis was emphasized in order to include as many relevant studies as possible); (3) ICH recurrence was defined as a new symptomatic ICH confirmed by imaging after the index ICH; (4) studies reporting CAA-ICH recurrence rates or any risk factor odds ratio (ORs), relative risk ratio (RRs), hazard ratio (HRs), 95% confidence interval (CIs), and equivalent data; (5) retrospective or prospective English original studies.

The following studies were excluded: (1) studies that did not provide the prevalence of CAA-ICH recurrence or could not obtain effect sizes for any risk factor for CAA-ICH recurrence; (2) studies with baseline and follow-up numbers of patients with CAA-ICH <30; (3) studies reporting results from overlapping patient cohorts were included after discussion by two authors, with priority given to studies providing additional or more detailed information; and (4) reviews, letters, abstracts of meetings, Supplementary material, case reports, and case series studies.

### 2.3. Data extraction and quality assessment

We used a standard pre-extraction table to extract the data required for this meta-analysis and further revised the table based on the preliminary extraction results. Two authors independently extracted the following information: first author, year of publication, study design, geographic region, sample characteristics (e.g., sample size, mean age or range, male proportion, and proportion of defined and probable patients with CAA), duration of follow-up, and number of relapses/total number of follow-ups (Table 1). If multiple articles were published on the same cohort, then the most informative report was included. When a follow-up study included multiple time points, we included the data for the longest follow-up time. We only counted the risk factors that were studied two or more times. In addition, we extracted the necessary imaging methods at baseline, diagnostic criteria for CAA, starting point of the study and relevant risk factors (Table 2).

**Table 1:** Characteristics of all included studies.

**References**	**Study year**	**Study design**	**Multicenter (Y/N)**	**Region (country)**	**Age, years; male ratio**	**Definite or probable CAA (%)**	**Follow-up**	**CAA-ICH cases (R/N)**	**NOS scores**
Yanagawa et al. (21)	2010–2019	Retrospective	N	Asia (Japan)	78 (57–89); 32%	100%	1, month	12/50	7
Koemans et al. (22)	1994–2012	Prospective	N	America (USA)	Mean:76; 49%	40.8%	71.2 (44.7–88.6), months	91/370	9
Yang et al. (23)	NA	Prospective	Y	Asia (China)	74 (66–80); 57.9%	25.4%	90, days	2 weeks: 10/197	9
								90 days: 17/185	
Goeldlin et al. (24)	2014–2019	Retrospective	Y	Europe (Switzerland)	77.1 ± 8.2; 43.8%	NR	3, months	14/185	8
Garg et al. (25)	2016–2018	Retrospective	Y	America (USA)	77.2 ± 8.2; 46.1%	NR	181.4 ± 106.4, days	369/7,857	9
Che et al. (26)	2014–2020	Prospective	N	Asia (China)	71 (64.5–79.0); 53.9%	NR	19.0 (12.0–26.5), months	1 year: 8/141	9
								long term: 12/141	
Xu et al. (27)	2012–2019	Prospective	Y	Asia (China)	71.0 ± 9.9; 53.2%	NR	1, year	5/60	8
Xia et al. (28)/Cheng et al. (29)	2014–2018	Prospective	Y	Asia (China)	70.1 ± 9.1; 73.5%	100%	2.4 (1.3–4.0), years	19/68	9
Tsai et al. (30)	2014–2018	Retrospective	N	Asia (China/Taiwan)	73.3 ± 13.8; 46.9%	NR	26 (11–34), months	8/32	7
Pinho et al. (31)	2014–2017	Retrospective	N	Europe (Germany)	70.9 ± 10.2; 50%	85.4%	24 (14–43), months	13/48	9
Li et al. (32)	1997–2012	Prospective	N	America (USA)	76.4 ± 8.7; 45.5%	59.4%	2.66 (0.89–5.20), years	49/244	9
Raposo et al. (33)	1997–2014	Prospective	N	America (USA)	76.2 ± 8.7; 47.5%	58.6%	28.3 (7.2–57.0), months	54/261	9
Pasi et al. (34)	2003–2012	Prospective	N	America (USA)	74.9 ± 9.3; 49.7%	100%	4.1 ± 3.2, years	52/150	9
Charidimou et al. (35)/Charidimou et al. (36)	2003–2012	Prospective	N	America (USA)	70.3 ± 11.1; 52.9%	61.7%	2.6 (0.9–5.1), years	58/240	9
Xia et al. (37)	2012–2015	Prospective	N	Asia (China)	Mean: 70.6; 66.3%	63.2%	Mean: 14.47, months	27/83	9
Boulouis et al. (38)	1994–2012	Prospective	N	America (USA)	73 ± 10.7; 47%	61.1%	2.8 (0.9–5.4), years	56/229	9
Roongpiboonsopit et al. (39)	1994–2012	Prospective	N	America (USA)	76.44 ± 8.83; 47.6%	60.3%	28.1 (6.59–56.03), months	6 months: 21/292	9
								28 months: 55/292	
Koo et al. (40)	2005–2013	Retrospective	N	Asia (Korea)	Probable: 70 ± 9.4/Possible: 69.8 ± 8.1; 56%	67.1%	Mean: 35.7 (range: 1–121), months	11/85	8
Yeh et al. (41)	1995–2013	Prospective	N	Asia (China/Taiwan)	74 (66–81); 51.0%	NR	5.5 ± 5.3, years	1 year: 5.6%	8
								5 years: 13.9%	
								10 years: 25.8%	
								15 years: 35.6%	
van Etten et al. (42)	1993–2012	Prospective	N	America (USA)	73.6 ± 9; 51%	100%	5.3 ± 3.8, years	86/240	9
Charidimou et al. (43)	2002–2010	Retrospective	Y	Europe (UK)	71.3 ± 8; 52.1%	79.7%	24 (9–44), months	20/104	8
Biffi et al. (44)	2003–2009	Prospective	N	America (USA)	74.4 ± 7.8; 56.2%	100%	34.3 (15.1–57.6), months	27/64	9
Domingues-Montanari et al. (45)	2003–2009	Prospective	N	Europe (Spain)	74.7 ± 7.0; 52.9%	75.4%	36, months	18/60	9
Biffi et al. (46)	1994–2006	Prospective	N	America (USA)	72.5 ± 8.2; 56.2%	NR	34.3 (15.1–57.6), months	29/104	8
Petridis et al. (47)	1991–2004	Retrospective	N	Europe (Germany)	NR; 40%	100%	NR	22/99	8
Lzumihara et al. (48)	1987–1997	Retrospective	Y	Asia (Japan)	NR; 42.5%	100%	NR	12/40	7
Greenberg et al. (49)	1994–2002	Prospective	N	America (USA)	NR; 51%	NR	20.8 ± 16.7, months	27/94	9
O'Donnell et al. (50)	1994–1998	Prospective	N	America (USA)	75.4 ± 8.4; 52.1%	69%	23.9 ± 14.8, months	19/71	9

Data presented as mean ± standard deviation (SD), median (range), n (%) or otherwise stated.

CAA, cerebral amyloid angiopathy; ICH, Intracerebral hemorrhage; NOS, Newcastle-Ottawa Scale; NR, non-reported.

**Table 2:** Risk factors of included studies.

**References**	**Necessary imaging at baseline**	**Diagnostic criteria for CAA**	**Inception point**	**Recurrence rate**	**Extraction of risk factors**	
					**Univariate analyses**	**Multivariate analyses**
Yang et al. (23)	CT	Original Boston criteria	Discharged	9.2%	F2, F4, F5, F9	F1, F3, F6, F7, F8, F10, F11
Garg et al. (25)	NR	ICD-10 code I68.0	Discharged	4.7%	F3, F4, F5	F1, F2
Che et al. (26)	CT	Modified Boston criteria	1, week	8.5%	NA	F1, F9, F10
Xia et al. (28)	CT/MRI	Modified Boston criteria	1, month	27.9%	F1, F2, F3, F4, F9, F12, F14	NA
Pinho et al. (31)	MRI	Modified Boston criteria	Discharged	27.1%	F2, F3, F5, F7, F8, F9, F12	F1, F15
Li et al. (32)	CT and MRI	Modified Boston criteria	Discharged	20.1%	NA	F1, F6, F7, F8, F11, F12, F13
Raposo et al. (33)	MRI	Original Boston criteria	Discharged	20.7%	NA	F1, F6
Cheng et al. (29)	CT and MRI	Modified Boston criteria	1, month	27.9%	F13, F15	F16
Charidimou et al. (35)	MRI	Original Boston criteria	1, month	24.2%	F2, F12	F1, F12, F13, F15
Charidimou et al. (36)	MRI	Original Boston criteria	1, month	24.2%	NA	F6
Boulouis et al. (38)	MRI	Original Boston criteria	1, month	24.5%	F2, F6, F5, F12, F15	F1, F3, F4, F13
Roongpiboonsopit et al. (39)	CT and MRI	Original Boston criteria	Discharged	18.8%	NA	F1, F6, F11, F13
Koo et al. (40)	MRI	Original Boston criteria	1, week	12.9%	NA	F12, F13, F14
Biffi et al. (46)	CT and MRI	Original Boston criteria	3, months	27.9%	NA	F6, F8, F12, F15
Lzumihara et al. (48)	NR	Histological diagnosis	NA	30%	NA	F3
O'Donnell et al. (50)	MRI	Original Boston criteria	Discharged	26.8%	F1, F2, F3, F4, F5	F6

CT, computed tomography; ICD-10, Tenth Revision diagnosis code; MRI, magnetic resonance imaging; NA, Not available.

Risk Factors: F1, Age; F2, Gender; F3, Hypertension; F4, Diabetes; F5, Dyslipidemia; F6, Previous ICH; F7, Anticoagulation; F8, Antiplatelet therapy; F9, Baseline ICH volume; F10, IVH presence; F11, cSAH presence; F12, Lobar CMBs; F13, cSS; F14, CSO-PVS; F15, WMH volume; F16, Total MRI burden of SVD.

We preferentially included the results from analyses adjusted for multiple factors to extract OR (95% CI) in the models with the largest number of variables. In the absence of multifactor analysis, the results of the single-factor analysis were included. The Newcastle-Ottawa Scale (NOS) recommended by the Cochrane manual was used to assess the quality of the included studies. It consists of eight items and three components: selection, comparability, and outcomes (cohort studies) or exposure (case-control studies). The highest quality studies scored as high as 9. A study with an NOS score ≥7 indicated high quality. The NOS scores of each study are presented in Supplementary Table 3. Two authors independently evaluated the quality of each included study, and disputes were fully discussed with senior investigators to resolve any differences in the data extraction or quality assessment.

### 2.4. Statistical analysis

STATA/SE 16.0 was used for all statistical analyses and the significance level was set at 0.05. The “metan” function of STATA/SE 16.0 was used to estimate the recurrence rate of CAA-ICH and the pooled ORs (95% CI) of associated risk factors. Heterogeneity in all included studies was assessed and quantified using Cochrane Q and I^2^ statistics, respectively. If I^2^ > 50%, the heterogeneity of the included studies was considered significant, and a random-effects model was used to summarize the results. If I^2^ ≤ 50%, the fixed-effects model was used. Heterogeneity was also addressed by meta-regression and subgroup analysis. Sensitivity analysis was used to assess the stability of the merger effect. Funnel plot and Egger's test were used to estimate publication bias.

### 2.5. Studies quality

The quality of evidence was assessed using the Grading of Recommendations, Assessments, Developments and Evaluations (GRADE) system (51), which resulted in a high, moderate, low, or very low level of evidence. Disagreements encountered during the assessment process could be resolved by mutual consultation between the two authors.

## 3. Results

### 3.1. Study selection and study characteristics

**Figure 1:**
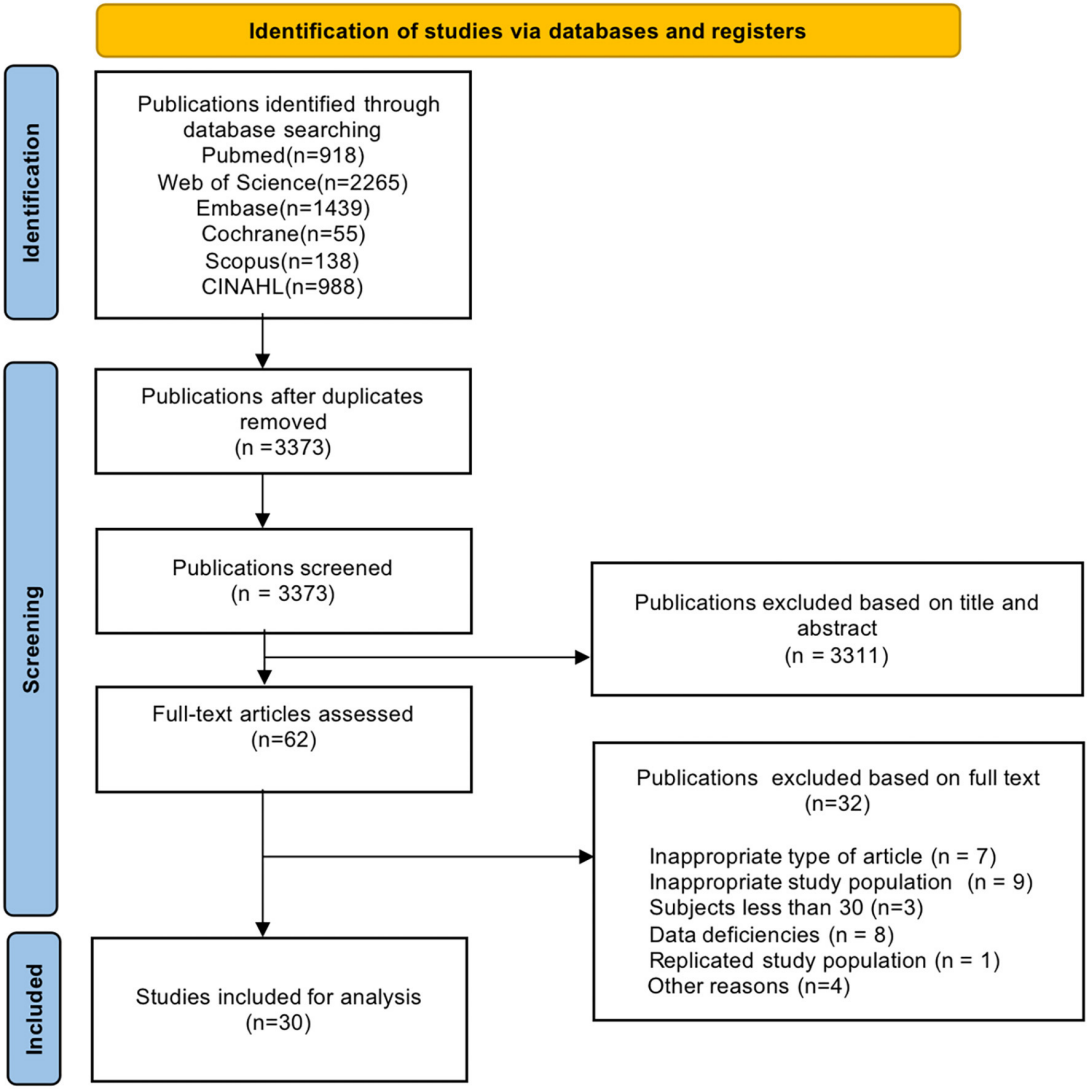
The PRIMSA flow diagram of this study.

After conducting the literature search, we initially identified 5,803 articles, including 918 in PubMed, 2,265 in Web of Science, 1,439 in EMBASE, 55 in the Cochrane Library, 138 in Scopus, and 988 in CINAHL. After excluding duplicate studies, 3,311 unrelated studies among the 3,373 were further excluded based on their title/abstract. The remaining 62 studies were eligible for full-text review, of which 30 met the inclusion and exclusion criteria and were included in the meta-analysis (21–50). Figure 1 presents a flowchart of the literature selection process.

Among the included studies, 28 cohort studies (21–24, 26–30, 32–50) and two case-control studies (25, 31) with publication years ranging from 2000–2023, and baseline characteristics are presented in Table 1. Of these, 11 were from Asia (21, 23, 26–30, 37, 40, 41, 48), five from Europe (24, 31, 43, 45, 47), and 14 from the Americas (22, 25, 32–36, 38, 39, 42, 44, 46, 49, 50). The sample size of the included studies varied from 32 (30) to 7,857 (26), and the mean follow-up duration varied from 1 month (21) to 71.2 months (22). Each study reported the number of symptomatic ICH relapses during the study period and the total study population. Sixteen studies were used to further explore the risk factors associated with CAA-ICH recurrence (23, 25, 26, 28, 29, 31–33, 35, 36, 38–40, 46, 48, 50) (Table 2). In addition, the NOS scores for the quality of the included studies ranged from 7 to 9 points, indicating high quality (Supplementary Table 3).

Finally, we analyzed 16 potential risk factors for CAA-ICH recurrence, including general demographic characteristics: age, sex; previous history of hypertension, diabetes, dyslipidemia, previous ICH (i.e., hemorrhagic stroke before the index lobar hemorrhage); use of antithrombotic drugs: antiplatelet or anticoagulation therapy; imaging manifestations visible on CT: baseline ICH volume, presence of subarachnoid hemorrhage (cSAH), presence of intraventricular hemorrhage (IVH), MRI-based imaging markers including lobar CMBs, cSS, CSO-PVS, WMH volume (WMH volume refers to the total volume of WMH measured on liquid-attenuated inversion recovery MRI using a computer-assisted measurement method), and total MRI burden of SVD.

### 3.2. Recurrence rate

**Figure 2:**
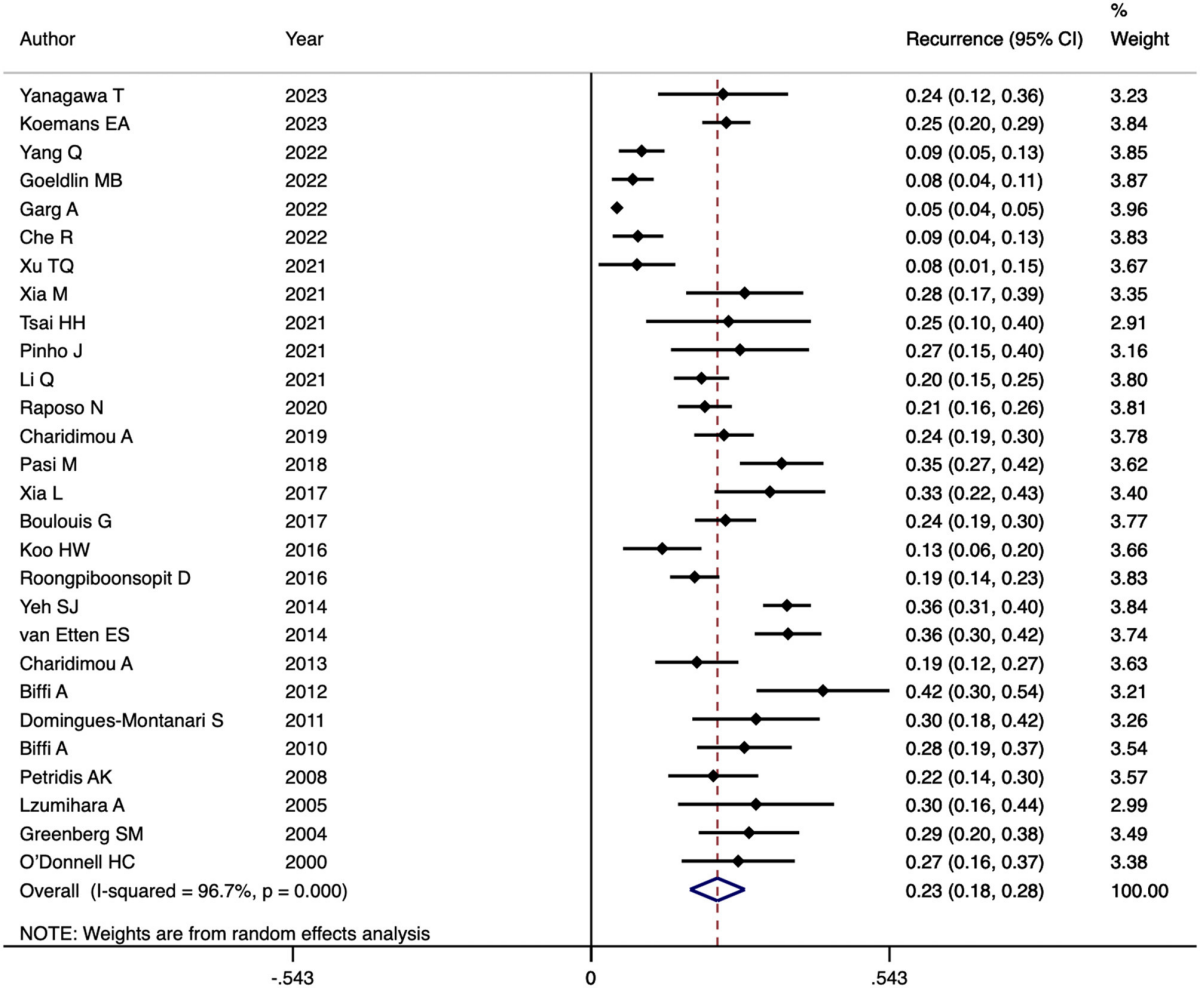
Forest plot of CAA-ICH recurrence rate.

Twenty-eight studies were included to calculate the recurrence rate of CAA-ICH. In the included studies, CAA-ICH recurrence rates ranged from 4.7 to 42.2%, with pooled recurrence rates of 23% (95% CI:18–28%) and significant heterogeneity (I^2^ = 96.7%; *P* < 0.001) (Figure 2).

The meta-regression analysis showed that age (*P* = 0.129), gender (*P* = 0.362), hypertension (*P* = 0.857) and diabetes (*P* = 0.84) were not sources of heterogeneity.

Subgroup analysis by region: The recurrence rate of CAA-ICH in Asian population was 21% (95% CI: 13–29%; I^2^ = 92.6%; *P* < 0.001; *N* = 10); in the Americas, the recurrence rate was 25% (95% CI: 17–33%; I^2^ = 97.7%; *P* < 0.001; *N* = 13); in Europe, the recurrence rate was 20% (95% CI: 11–30%; I^2^ = 85.8%; *P* < 0.001; *N* = 5) (Supplementary Figure 1A).

Subgroup analysis based on follow-up duration: The recurrence rate at 1 year or less was 8% (95% CI: 5–12%; I^2^ = 77.2%; *P* = 0.002; *N* = 5), and the recurrence rate after 1 year was 24% (95% CI: 17–31%; I^2^ = 84.4%; *P* < 0.001; *N* = 21). The subgroup analysis showed that region and follow-up duration were not sources of study heterogeneity in CAA-ICH recurrence rates (Supplementary Figure 1B).

### 3.3. Risk factors for CAA-ICH recurrence

#### 3.3.1. General demographic characteristics

Eleven studies investigated the association between age and CAA-ICH recurrence. Results were summarized using a random effects model (OR = 1.02; 95% CI: 0.99–1.05; *P* = 0.19; I^2^ = 70.4%; *P* < 0.001). The pooled results of seven studies involving the relationship between sex and CAA-ICH recurrence showed no significant association between sex and CAA-ICH recurrence (OR = 0.95; 95% CI: 0.77–1.17; *P* = 0.62; I^2^ = 0%; *P* = 0.99) (Supplementary Table 4).

#### 3.3.2. Medical history

**Figure 3:**
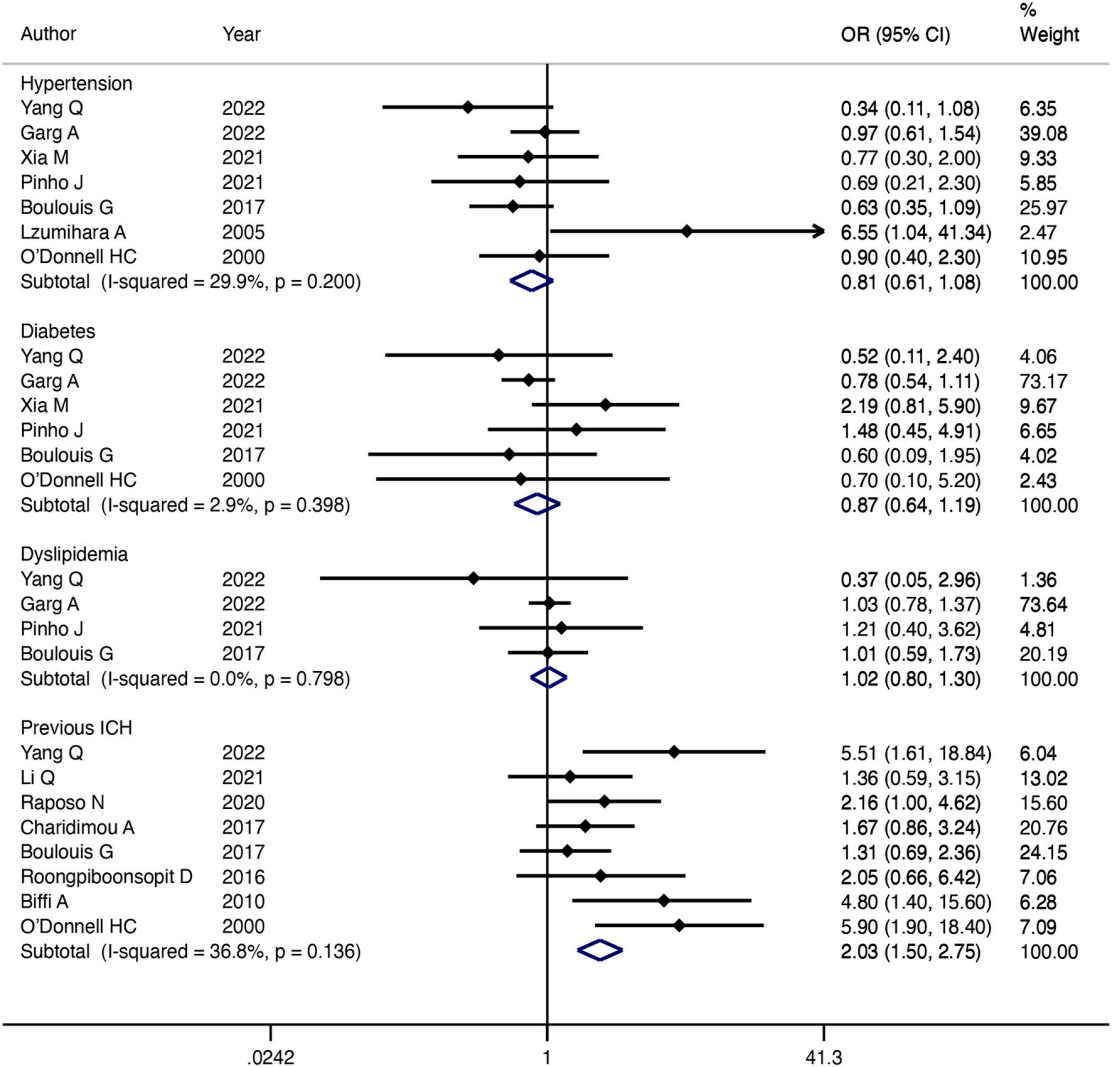
Forest plot of the association between medical history and CAA-ICH recurrence.

The association between hypertension and CAA-ICH recurrence was analyzed in seven studies. The pooled results using a fixed-effect model of 7 studies suggested that there was no significant difference in recurrence rate between patients with hypertension and patients without hypertension (OR = 0.81; 95% CI: 0.61–1.08; *P* = 0.15; I^2^ = 29.9%; *P* = 0.20) (Figure 3). Six studies analyzed the influence of diabetes mellitus on CAA-ICH recurrence. Our pooled results of six studies using a fixed-effect model indicated that there was no significant relationship between diabetes mellitus and CAA-ICH recurrence, with no heterogeneity among included studies (OR = 0.87; 95% CI: 0.64–1.19; *P* = 0.39; I^2^ = 2.9%; *P* = 0.40) (Figure 3). Four studies reported an association between dyslipidemia and the recurrence of CAA-ICH. Our pooled results of four studies indicated that there was no significant relationship between dyslipidemia and CAA-ICH recurrence (OR = 1.02; 95% CI: 0.80–1.30; *P* = 0.88; I^2^ = 0%; *P* = 0.80) (Figure 3). Eight studies analyzed the influence of previous ICH on CAA-ICH recurrence. The pooled evidence with a fixed-effect model suggested that patients with CAA-ICH with previous ICH were more susceptible to relapse (OR = 2.03; 95% CI: 1.5–2.75; *P* < 0.001; I^2^ = 36.8%; *P* = 0.14) (Figure 3).

#### 3.3.3. Use of antithrombotic drugs

The impact of anticoagulation therapy on CAA-ICH recurrence has been reported in three studies. We used a random effects model to summarize the results, which showed that there was no significant relationship between anticoagulation and CAA-ICH recurrence (OR = 1.72; 95% CI: 0.16–18.13; *P* = 0.651; I^2^ = 74.2%; *P* = 0.02) (Supplementary Table 4).

Four studies analyzed the influence of antiplatelet therapy on CAA-ICH recurrence. Our analysis results suggested no significant relationship between antiplatelet therapy and the recurrence of CAA-ICH. We used a random-effect model because of the significant heterogeneity observed among these studies (OR = 1.66; 95% CI: 0.66–4.15; *P* = 0.28; I^2^ = 69.7%; *P* = 0.02) (Supplementary Table 4).

#### 3.3.4. ICH imaging manifestations at baseline based on CT

**Figure 4:**
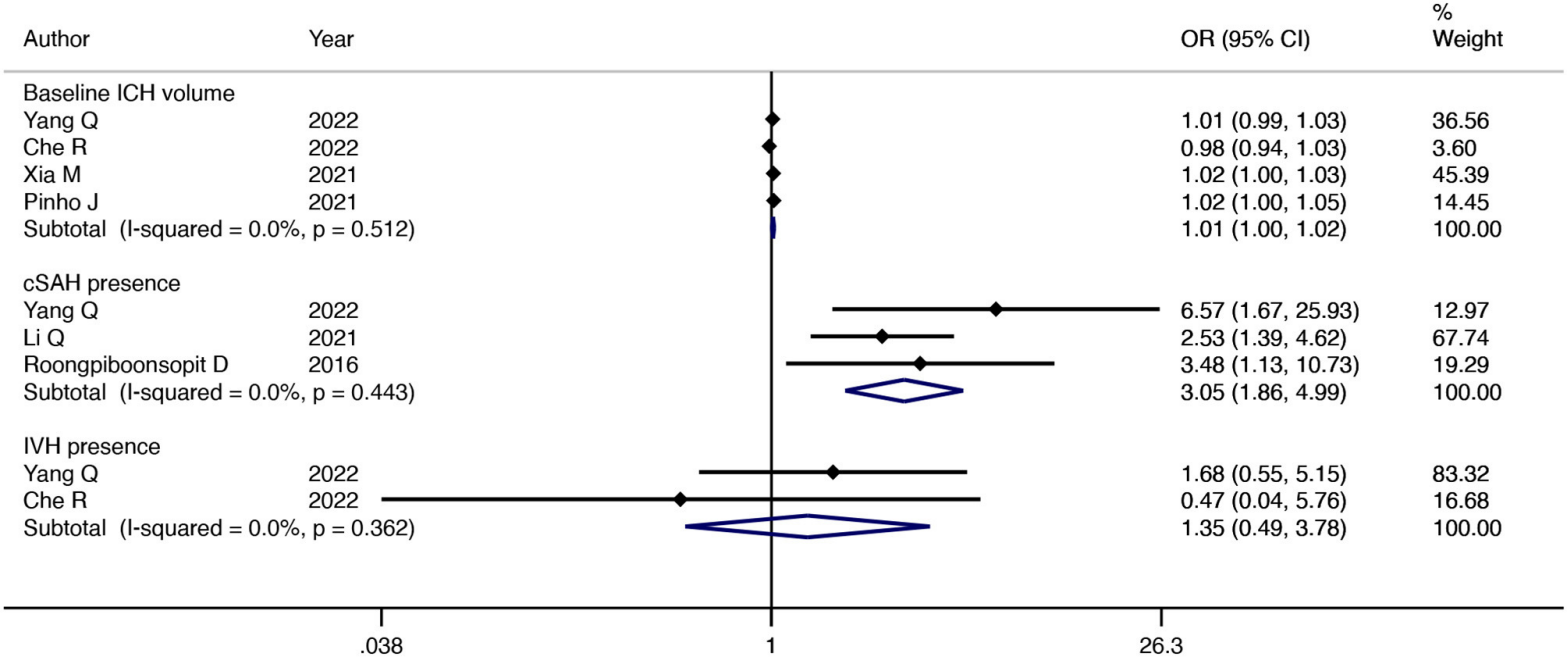
Forest plot showing the association between ICH imaging manifestations at baseline based on CT and CAA-ICH recurrence.

Four studies reported an association between baseline ICH volume and CAA-ICH recurrence. The pooled results with a fixed-effects model showed that the larger the baseline ICH volume, the higher the risk of CAA-ICH recurrence (OR = 1.01; 95% CI: 1–1.02; *P* = 0.004; I^2^ = 0%; *P* = 0.51) (Figure 4). A fixed-effect model was used to summarize the results of two studies and showed that there was no significant association between the presence of IVH and CAA-ICH recurrence (OR = 1.35; 95% CI: 0.49–3.78; *P* = 0.56; I^2^ = 0%; *P* = 0.36) (Figure 4). The pooled results with a fixed-effect model of three studies suggested that patients with CAA-ICH along with cSAH were more susceptible to relapse (OR = 3.05; 95% CI: 1.86–4.99; *P* < 0.001; I^2^ = 0%; *P* = 0.44) (Figure 4).

#### 3.3.5. MRI imaging markers and total MRI burden of SVD

**Figure 5:**
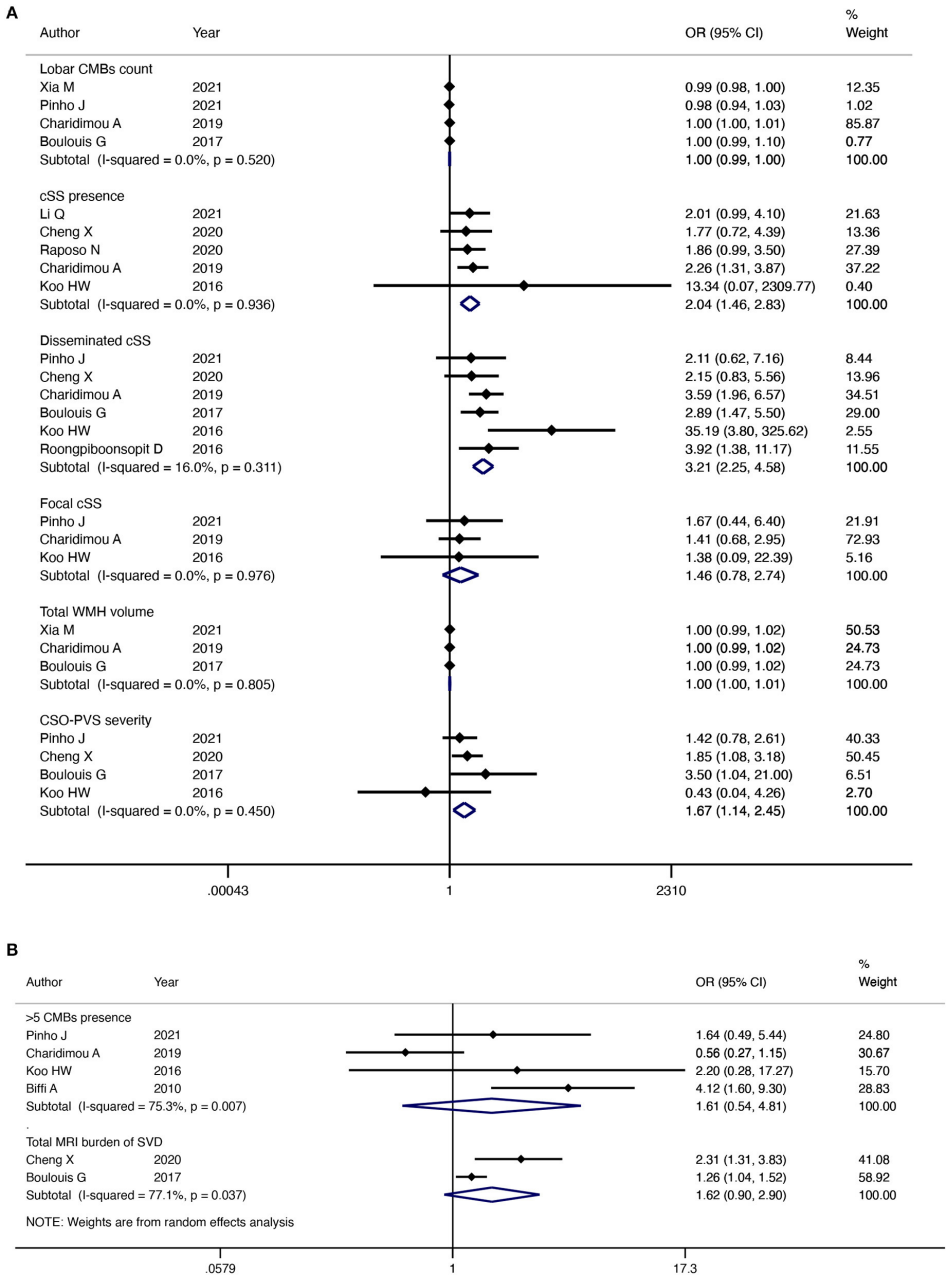
Forest plot showing the association between MRI imaging markers and CAA-ICH recurrence. I^2^ ≤ 50%, the fixed effects model was used **(A)**, I^2^> 50%, the random effects model was used **(B)**.

Five studies investigated the association between lobar CMBs presence and CAA-ICH recurrence. Our pooled results of five studies indicated that there was no significant relationship between lobar CMBs count and CAA-ICH recurrence, with no heterogeneity among included studies (OR = 1.00; 95% CI: 0.99–1.00; *P* = 0.57; I^2^ = 0%; *P* = 0.52) (Figure 5A). In addition, the pooled results of four studies showed that lobar CMBs>5 was not significantly associated with CAA-ICH recurrence (OR = 1.61; 95% CI: 0.54–4.81; *P* = 0.39; I^2^ = 75.3%; *P* = 0.007) (Figure 5B).

Five studies reported an association between the presence of cSS and recurrence of CAA-ICH. The pooled evidence with a fixed-effect model indicated that patients with CAA-ICH and cSS were more susceptible to relapse (OR = 2.04; 95% CI: 1.46–2.83; *P* < 0.001; I^2^ = 0%; *P* = 0.94) (Figure 5A). Six studies investigated the association between disseminated cSS and CAA-ICH recurrence separately. The pooled evidence indicated that disseminated cSS was highly correlated with the recurrence of CAA-ICH (OR = 3.21; 95% CI: 2.25–4.58; *P* < 0.001; I^2^ = 16%; *P* = 0.31) (Figure 5A). However, the pooled results of the three studies suggested that there was no significant relationship between focal cSS and CAA-ICH recurrence (OR = 1.46; 95% CI: 0.78–2.74; *P* = 0.24; I^2^ = 0%; *P* = 0.98) (Figure 5A). As no significant heterogeneity was found, a fixed-effects model was used in both cases.

Three studies analyzed the relationship between WMH volume and CAA-ICH recurrence, and pooled results with a fixed-effect model showed no significant association between them (OR = 1.00; 95% CI: 1.00–1.01; *P* = 0.52; I^2^ = 0%; *P* = 0.45) (Figure 5A).

Four studies analyzed the association between CSO-PVS and CAA-ICH recurrence. The pooled evidence of four studies with a fixed-effect model suggested that the risk of recurrence of CAA-ICH was positively associated with the severity of CSO-PVS (OR = 1.67; 95% CI: 1.14–2.45; *P* = 0.009; I^2^ = 0%; *P* = 0.45) (Figure 5A).

The association between the total MRI burden of SVD and CAA-ICH recurrence has been reported in two studies. Our pooled results of two studies indicated that there was no significant relationship between the total MRI burden of SVD and the risk of CAA-ICH recurrence (OR = 1.62; 95% CI: 0.90–2.90; *P* = 0.11; I^2^ = 77.1%; *P* = 0.04) (Figure 5B). A random-effects model was used because significant heterogeneity was detected between the studies.

### 3.4. Sensitivity analysis and publication bias

**Figure 6:**
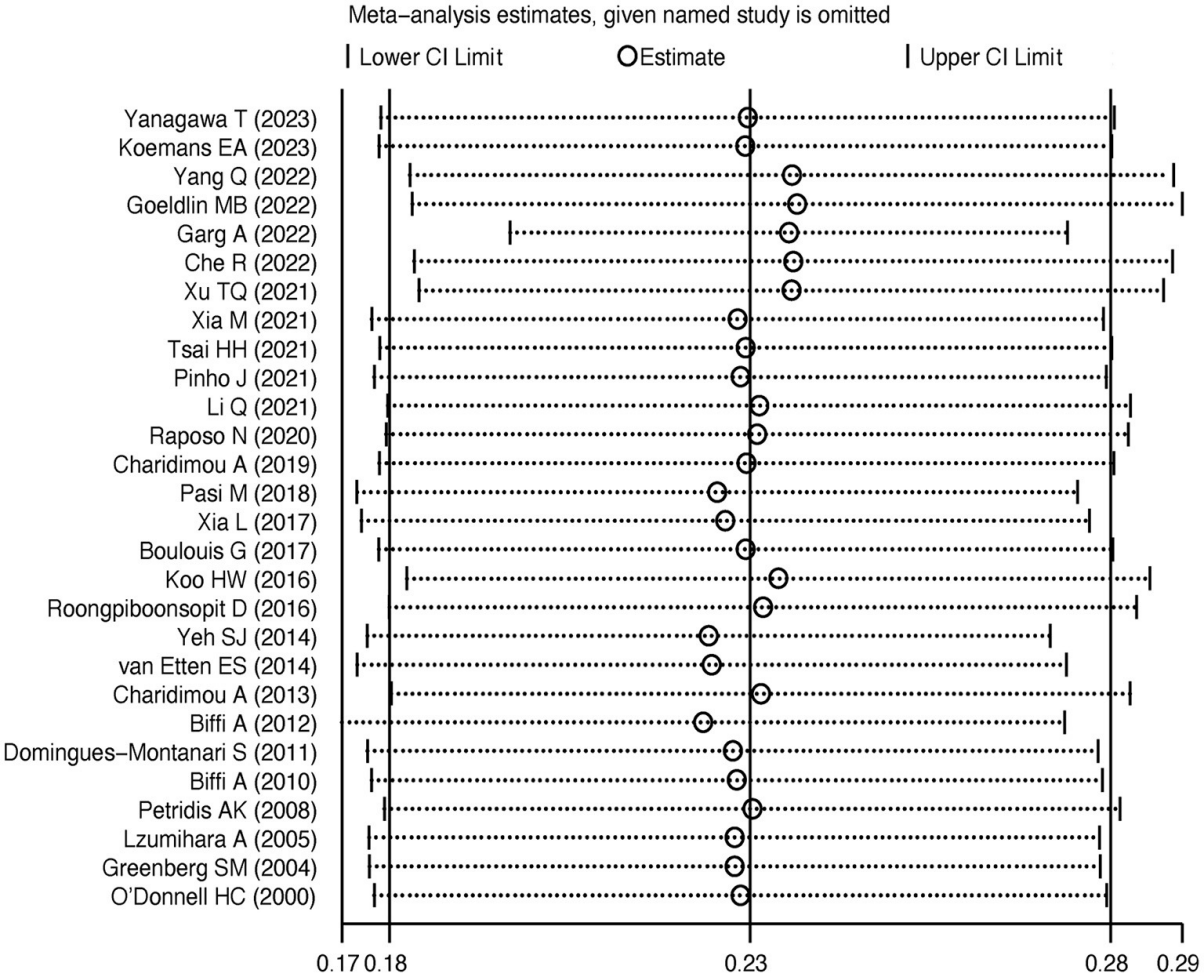
Sensitivity analysis examining the robustness of the results.

**Figure 7:**
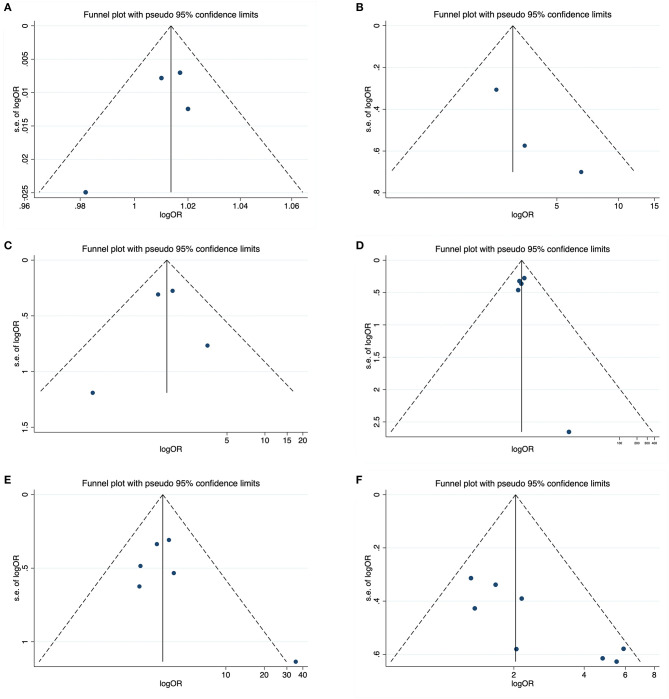
Funnel plots about potential publication bias for six recurrence risk factors including baseline ICH volume **(A)**, cSAH presence **(B)**, CSO-PVS **(C)**, cSS presence **(D)**, disseminated cSS **(E)**, and previous ICH **(F)**.

In sensitivity analysis, no individual study significantly influenced the pooled CAA-ICH recurrence rate (Figure 6). The funnel plots visually assess publication bias and the horizontal line represents the summary effect estimates. The funnel plots and Egger's tests of relapse risk factors, except for previous ICH (*P* = 0.008) (Figure 7F), showed no statistically significant publication bias for other risk factors, including baseline ICH volume (*P* = 0.364) (Figure 7A), cSAH presence (*P* = 0.224) (Figure 7B), CSO-PVS (*P* = 0.798) (Figure 7C), cSS presence (*P* = 0.343) (Figure 7D) and disseminated cSS (*P* = 0.333) (Figure 7E).

### 3.5. Rating the quality of evidence

**Figure 8:**
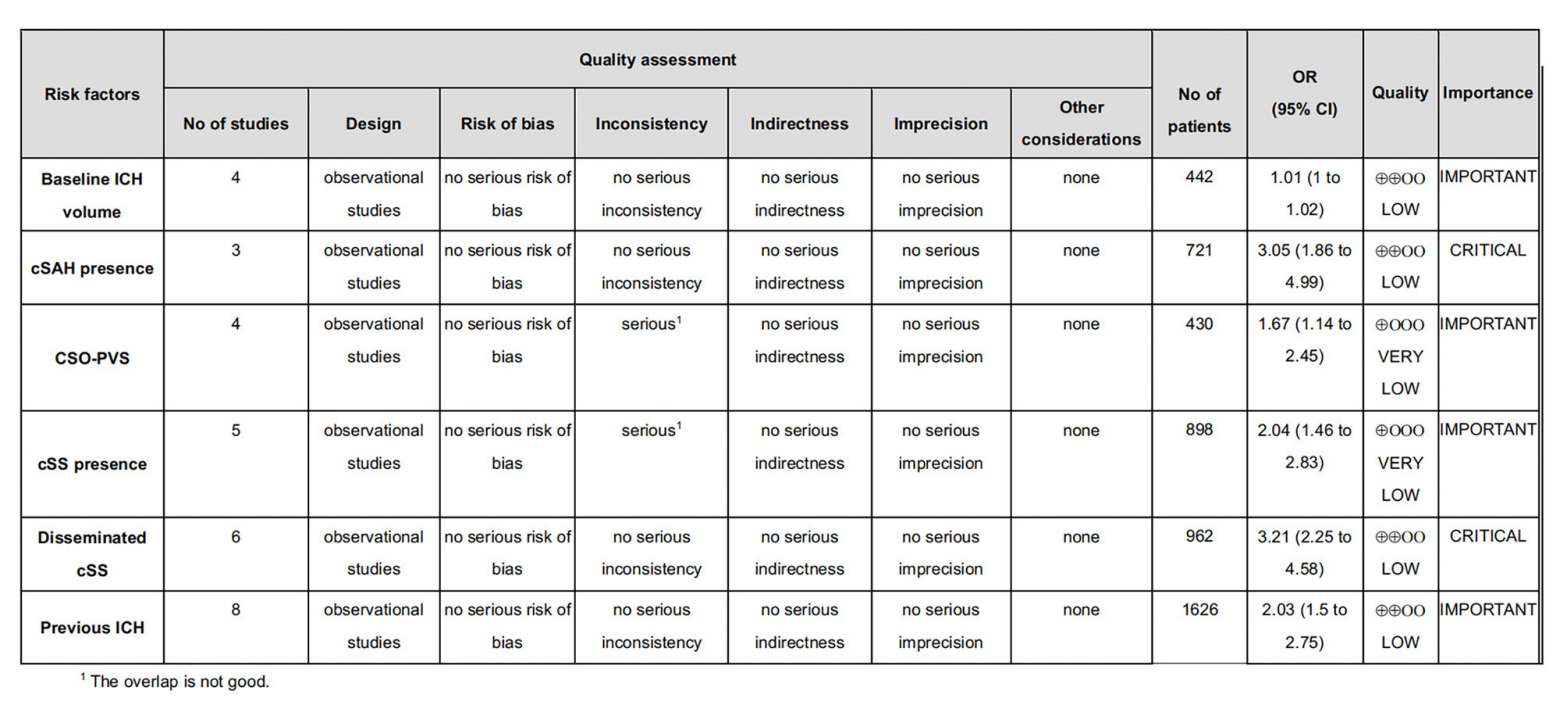
Assessment of study quality using GRADE.

According to the GRADE scores, the strength of evidence for the six risk factors mentioned above ranged between very low and low. All studies reported outcome indicators directly. The reason for the downgrade is shown in Figure 8.

## 4. Discussion

Our study confirmed a high recurrence rate of CAA-ICH. However, the significant heterogeneity among the included studies may have limited the reliability of the findings. Vascular risk factors, region and follow-up duration were not sources of heterogeneity. Furthermore, our study found that the risk factors associated with the recurrence of CAA-ICH included previous ICH, baseline ICH volume, presence of cSAH, cSS (especially if disseminated), and the severity of CSO-PVS (*P* < 0.05). However, the associations between age, sex, hypertension, diabetes, dyslipidemia, anticoagulation or antiplatelet therapy, presence of IVH, lobar CMBs, WMH volume, total MRI burden of SVD (P>0.05), and CAA-ICH recurrence require further investigation.

Our study showed that cSAH on CT, and cSS (especially if disseminated) and CSO-PVS on MRI are significant neuroimaging markers associated with CAA-ICH recurrence. They can serve as baseline predictors of ICH to identify patients at high risk for recurrence. The generation of cSS is attributed to the excessive deposition of amyloid-β, which leads to increased brittleness of the cortical or pia meningeal blood vessels (52). This brittleness can cause vessel rupture, resulting in chronic bleeding, and the deposition of hemosiderin and other blood decomposition substances in the superficial cortical surface and subarachnoid space (53). The incidence of cSS is high in patients with CAA-ICH but not in those with other types of ICH (54). According to the modified Boston criteria, the cSS is an important, clinically relevant imaging marker for the diagnosis of CAA (55). Some studies have found that the site of subsequent substantial cerebral hemorrhage in patients with CAA is often the same as the site of the initial cSS (56). A previous meta-analysis has also confirmed that cSS strongly predicts increased risk of recurrent lobar hemorrhage (35). Our study also showed that patients with more severe cSS (disseminated cSS: affecting three brain sulci and more) had a higher risk of future independent bleeding.

Similar to cSS, the mechanism by which cSAH can predict the recurrence of CAA-ICH may be related to the increased brittleness of blood vessels caused by the high deposition of amyloid proteins in the pia, leading to rupture. cSS and cSAH are likely two different imaging markers in a single pathological process (57). Focal convex linear SAH (acute cSAH) marks acute bleeding events and chronic cerebral sulci blood degradation products (cSS) mark previous episodes of cSAH (58). Previous studies have confirmed that the presence of MRI-detected cSHA is closely associated with the recurrence of CAA-ICH (54, 59). Specifically, adjacent cSAH is often associated with acute lobar ICH, which may extend from or develop directly from lobar ICH (60). This indicates that the area near the hematoma is more susceptible compared to distant areas. Sometimes, cSAH is clinically characterized by mild or asymptomatic symptoms that are easy to miss; however, its frequent onset eventually leads to symptomatic ICH (57). Owing to the limited number of available studies, in our study, cSAH was based on CT scans. CT scan is widely used in most medical institutions for the evaluation of acute ICH. Therefore, the discovery of CT-based cSAH has a greater advantage in determining early ICH recurrence.

The PVS is defined as a fluid-filled space that acts as a pre-lymphatic system for removing substances (40, 61). Owing to aging, the deterioration of vascular conditions, and other factors, the deposited amyloid-β obstructs the function of the original cleaning substance of the PVS, causing the expansion of the PVS to further hinder the drainage of soluble amyloid-β, leading to arterial load rupture and ICH (62). High CSO-PVS have been shown to be closely related to cSS and are highly prevalent in sporadic CAA (63). They hold as much potential as cSS for both diagnosing CAA and predicting ICH recurrence.

In our study, the two other MRI-based SVD markers, lobar CMBs and WMH volume, were not significantly associated with CAA-ICH recurrence. A previous meta-analysis showed that the presence and number of lobar CMBs were associated with a high risk of ICH recurrence, especially when more than 10 CMBs were counted (64). Our pooled results showed that neither the presence of lobar CMBs >5 nor the number of lobar CMBs was significantly associated with the recurrence of CAA-ICH. This may be due to the fact that a smaller number of CMBs implies milder cerebral microangiopathy and is less predictive of rebleeding compared with lobar CMBs >10. In addition, the included studies contained different group-level data, which may have affected our pooled results. However, it has also been suggested that specific disease phenotypes (e.g., more bleeding or active disease selected by cSS) are more valuable in predicting CAA-ICH recurrence than the overall disease burden (lobar CMBs count and other SVD markers) (53), which is largely consistent with the results of our meta-analysis. The difficulty of detecting WMH by conventional MRI may explain the lack of correlation between WMH and ICH recurrence. Therefore, the predictive value of CMB and WMH for CAA-ICH should be studied using larger and more comprehensive imaging datasets. In the CT-based imaging findings, except for cSAH, the recurrence of CAA-ICH correlated with the baseline ICH volume, which reflects the severity of the index ICH; however, there was no significant correlation with the presence or absence of IVH.

The use of antithrombotic drugs in CAA-ICH survivors remains controversial. Previous studies have shown that avoiding anticoagulant use is a safer option for patients with CAA prone to recurrent ICH (65, 66). They suggested that patients with both non-valvular atrial fibrillation and CAA should consider alternative therapies (such as left atrial appendage occlusion) after bleeding or replace warfarin with a relatively safe vitamin K antagonist (67). Antiplatelet agents used in most ischemic strokes and transient ischemic attacks appear to have a better safety profile than anticoagulants (68). Although antiplatelet drugs do not directly increase the risk of bleeding, they are associated with an increased prevalence of lobar microbleeds, which further increases the risk of developing symptomatic ICH (69). Our meta-analysis showed that neither anticoagulant nor antiplatelet therapy was significantly associated with CAA-ICH recurrence, a conclusion consistent with an existing study (70). Ischemic lesions are common in patients with CAA (71). For survivors of CAA-ICH, the timing of discontinuation or re-initiation of antithrombotic therapy depends on whether the risk of bleeding or ischemia is higher. The decision made by the clinician after fully weighing the benefits and risks is particularly critical and of great significance for patients who require the continuous use of antithrombotic drugs. The safety of antithrombotic therapy in patients with CAA-ICH should be determined by studying a larger established population.

Patients with a history of hemorrhagic stroke are at a higher risk of rebleeding. However, there was no significant correlation between the history of other diseases and CAA-ICH recurrence. A previous study suggested that hypertension may be the only significant clinical factor affecting the recurrence of CAA-ICH (48, 72). However, our statistical results showed that a history of hypertension was not associated with the recurrence of CAA-ICH. Although patients with CAA-ICH may show clinical or pathological evidence of hypertension, hypertension-associated ICH primarily presents as arteriolar sclerosis and fibrinoid necrosis, which differ significantly from the characteristics and mechanism of CAA-ICH (73, 74). Therefore, hypertension may affect the prognosis of patients with CAA-ICH in other ways, which requires further investigation. However, there is currently no better treatment for CAA-ICH survivors. Therefore, strict blood pressure control is recommended.

This study has several strengths that contribute to its robustness and reliability. There are a limited number of studies specifically on CAA-ICH, and this study included as many studies as possible related to CAA-ICH recurrence. The study populations were from different geographic regions, and the studies spanned a long period of time. We screened for several risk factors for clinical and imaging manifestations that are strongly associated with ICH recurrence, which can be very helpful for clinicians in making decisions about disease assessment and medication. In addition, the study used a standardized study scoring tool and adhered to the relevant guidelines for systematic reviews and meta-analyses, which ensured the rigor and validity of the study.

Our study had several limitations. First, the number of included studies and the small sample size may have limited the reliability of our results for certain risk factors. Second, considering the limited literature available, we broadened the search scope to include all prospective or retrospective studies involving the CAA-ICH recurrence rate or any recurrence factors. The duration of follow-up varied widely among the included studies. These limitations prevent us from extending our conclusions to other populations. Therefore, large-scale, prospective, high-quality studies in different countries are necessary to confirm our findings.

In conclusion, our study confirmed a high recurrence rate of CAA-ICH. cSAH on CT, and cSS (especially if disseminated) and CSO-PVS on MRI were neuroimaging markers significantly associated with CAA-ICH recurrence. The onset of CAA-ICH usually occurs several times, and recurrence is partly related to the index ICH volume. Therefore, it should be highly valued and prevented after the initial bleeding event. Screening for effective baseline predictors can help develop rational prevention strategies to reduce the occurrence of rebleeding events and improve the prognosis of patients with CAA-ICH, which is currently the best option in the absence of specific treatment.

## Data availability statement

The original contributions presented in the study are included in the article/Supplementary material, further inquiries can be directed to the corresponding authors.

## Author contributions

XJ: Formal analysis, Writing—original draft, Software. MB: Formal analysis, Writing—original draft, Software. HZ: Data curation, Writing—review & editing. JX: Writing—review & editing, Supervision. LP: Methodology, Funding acquisition, Writing—review & editing. ZL: Funding acquisition, Methodology, Resources, Writing—review & editing.
