# Supplementary material for: Risk factors for recurrent cerebral amyloid angiopathy-related intracerebral hemorrhage
Source: Front Neurol. 2023 Nov 7;14:1265693. doi: 10.3389/fneur.2023.1265693 (PMC10661374; doi:10.3389/fneur.2023.1265693)
Supplement: Supplementary file 1 [file Data_Sheet_1.docx]

Supplementary Material

# Supplementary Tables and Figure

## Supplementary Tables

**Supplementary Table 1.** PRISMA checklist.

| **Section/topic** | **#** | **Checklist item** | **Reported on page #** |
| --- | --- | --- | --- |
| **TITLE** |  |  |  |
| Title | 1 | Identify the report as a systematic review, meta-analysis, or both. | 1 - 2 |
| **ABSTRACT** |  |  |  |
| Structured summary | 2 | Provide a structured summary including, as applicable: background; objectives; data sources; study eligibility criteria, participants, and interventions; study appraisal and synthesis methods; results; limitations; conclusions and implications of key findings; systematic review registration number. | 1 - 2 |
| **INTRODUCTION** |  |  |  |
| Rationale | 3 | Describe the rationale for the review in the context of what is already known. | 2 - 3 |
| Objectives | 4 | Provide an explicit statement of questions being addressed with reference to participants, interventions, comparisons, outcomes, and study design (PICOS). | 3 |
| **METHODS** |  |  |  |
| Protocol and registration | 5 | Indicate if a review protocol exists, if and where it can be accessed (e.g., Web address), and, if available, provide registration information including registration number. | 2 |
| Eligibility criteria | 6 | Specify study characteristics (e.g., PICOS, length of follow-up) and report characteristics (e.g., years considered, language, publication status) used as criteria for eligibility, giving rationale. | 3 |
| Information sources | 7 | Describe all information sources (e.g., databases with dates of coverage, contact with study authors to identify additional studies) in the search and date last searched. | 3 |
| Search | 8 | Present full electronic search strategy for at least one database, including any limits used, such that it could be repeated. | 3 |
| Study selection | 9 | State the process for selecting studies (i.e., screening, eligibility, included in systematic review, and, if applicable, included in the meta-analysis). | 3 |
| Data collection process | 10 | Describe method of data extraction from reports (e.g., piloted forms, independently, in duplicate) and any processes for obtaining and confirming data from investigators. | 3 - 4 |
| Data items | 11 | List and define all variables for which data were sought (e.g., PICOS, funding sources) and any assumptions and simplifications made. | 3 |
| Risk of bias in individual studies | 12 | Describe methods used for assessing risk of bias of individual studies (including specification of whether this was done at the study or outcome level), and how this information is to be used in any data synthesis. | 4 |
| Summary measures | 13 | State the principal summary measures (e.g., risk ratio, difference in means). | 3 |
| Synthesis of results | 14 | Describe the methods of handling data and combining results of studies, if done, including measures of consistency (e.g., *I^2^*) for each meta-analysis. | 4 |
| Risk of bias across studies | 15 | Specify any assessment of risk of bias that may affect the cumulative evidence (e.g., publication bias, selective reporting within studies). | 7 |
| Additional analyses | 16 | Describe methods of additional analyses (e.g., sensitivity or subgroup analyses, meta-regression), if done, indicating which were pre-specified. | 5, 7 |
| **RESULTS** |  |  |  |
| Study selection | 17 | Give numbers of studies screened, assessed for eligibility, and included in the review, with reasons for exclusions at each stage, ideally with a flow diagram. | 4 |
| Study characteristics | 18 | For each study, present characteristics for which data were extracted (e.g., study size, PICOS, follow-up period) and provide the citations. | 4 |
| Risk of bias within studies | 19 | Present data on risk of bias of each study and, if available, any outcome level assessment (see item 12). | 4 |
| Results of individual studies | 20 | For all outcomes considered (benefits or harms), present, for each study: (a) simple summary data for each intervention group (b) effect estimates and confidence intervals, ideally with a forest plot. | 5 - 7 |
| Synthesis of results | 21 | Present results of each meta-analysis done, including confidence intervals and measures of consistency. | 5 - 7 |
| Risk of bias across studies | 22 | Present results of any assessment of risk of bias across studies (see Item 15). | 7 |
| Additional analysis | 23 | Give results of additional analyses, if done (e.g., sensitivity or subgroup analyses, meta-regression [see Item 16]). | 5, 7 |
| **DISCUSSION** |  |  |  |
| Summary of evidence | 24 | Summarize the main findings including the strength of evidence for each main outcome; consider their relevance to key groups (e.g., healthcare providers, users, and policy makers). | 5 - 7 |
| Limitations | 25 | Discuss limitations at study and outcome level (e.g., risk of bias), and at review-level (e.g., incomplete retrieval of identified research, reporting bias). | 9 - 10 |
| Conclusions | 26 | Provide a general interpretation of the results in the context of other evidence, and implications for future research. | 10 |
| **FUNDING** |  |  |  |
| Funding | 27 | Describe sources of funding for the systematic review and other support (e.g., supply of data); role of funders for the systematic review. | 10 |

NA, not applicable.

**Supplementary Table 2.** Actual search strategies.

| ***PubMed Search Strategy*** | |
| --- | --- |
| #1 | ((((Cerebral Amyloid Angiopathy[MeSH Terms]) OR (Cerebral Amyloid Angiopathies[Title/Abstract])) OR (Congophilic Angiopathy[Title/Abstract])) OR (Congophilic Angiopathies[Title/Abstract])) OR (CAA[Title/Abstract]) |
| #2 | ((((((((((Cerebral Hemorrhage[MeSH Terms]) OR (Intracerebral Hemorrhage[Title/Abstract])) OR (Intracerebral Hemorrhages[Title/Abstract])) OR (Cerebral Hemorrhages[Title/Abstract])) OR (Cerebrum Hemorrhage[Title/Abstract])) OR (Cerebrum Hemorrhages[Title/Abstract])) OR (Cerebral Parenchymal Hemorrhage[Title/Abstract])) OR (Cerebral Parenchymal Hemorrhages[Title/Abstract])) OR (Cerebral Brain Hemorrhage[Title/Abstract])) OR (Cerebral Brain Hemorrhages[Title/Abstract])) OR (ICH[Title/Abstract]) |
| #3 | #1 AND #2 |
|  | |
| ***Embase Search Strategy*** | |
| #1 | caa:ti,ab,kw OR 'cerebral amyloid angiopathy':ti,ab,kw OR 'cerebral amyloid angiopathies':ti,ab,kw OR 'congophilic angiopathy':ti,ab,kw OR 'congophilic angiopathies':ti,ab,kw |
| #2 | ich:ti,ab,kw OR 'intracerebral hemorrhage':ti,ab,kw OR 'intracerebral hemorrhages':ti,ab,kw OR 'cerebral hemorrhage':ti,ab,kw OR 'cerebral hemorrhages':ti,ab,kw OR 'cerebrum hemorrhage':ti,ab,kw OR 'cerebrum hemorrhages':ti,ab,kw OR 'cerebral parenchymal hemorrhage':ti,ab,kw OR 'cerebral parenchymal hemorrhages':ti,ab,kw OR 'cerebral brain hemorrhage':ti,ab,kw OR 'cerebral brain hemorrhages':ti,ab,kw |
| #3 | #1 AND #2 |
|  | |
| ***Web of science Search Strategy*** | |
| #1 | ALL=(CAA OR Cerebral Amyloid Angiopathy OR Cerebral Amyloid Angiopathies OR Congophilic Angiopathy OR Congophilic Angiopathies) |
| #2 | ALL=(ICH OR Intracerebral Hemorrhage OR Intracerebral Hemorrhages OR Cerebral Hemorrhage OR Cerebral Hemorrhages OR Cerebrum Hemorrhage OR Cerebrum Hemorrhages OR Cerebral Parenchymal Hemorrhage OR Cerebral Parenchymal Hemorrhages OR Cerebral Brain Hemorrhage OR Cerebral Brain Hemorrhages) |
| #3 | **#1 AND #2** |
|  | |
| ***The Cochrane Library Search Strategy*** | |
| #1 | (CAA OR Cerebral Amyloid Angiopathy OR Cerebral Amyloid Angiopathies OR Congophilic Angiopathy OR Congophilic Angiopathies):ti,ab,kw |
| #2 | (ICH OR Intracerebral Hemorrhage OR Intracerebral Hemorrhages OR Cerebral Hemorrhage OR Cerebral Hemorrhages OR Cerebrum Hemorrhage OR Cerebrum Hemorrhages OR Cerebral Parenchymal Hemorrhage OR Cerebral Parenchymal Hemorrhages OR Cerebral Brain Hemorrhage OR Cerebral Brain Hemorrhages):ti,ab,kw |
| #3 | **#1 AND #2** |
|  | |
| ***Scopus Search Strategy*** | |
| #1 | ALL ( caa OR cerebral AND amyloid AND angiopathy OR cerebral AND amyloid AND angiopathies OR congophilic AND angiopathy OR congophilic AND angiopathies ) |
| #2 | ALL ( ich OR intracerebral AND hemorrhage OR intracerebral AND hemorrhages OR cerebral AND hemorrhage OR cerebral AND hemorrhages OR cerebrum AND hemorrhage OR cerebrum AND hemorrhages OR cerebral AND parenchymal AND hemorrhage OR cerebral AND parenchymal AND hemorrhages OR cerebral AND brain AND hemorrhage OR cerebral AND brain AND hemorrhages ) |
| #3 | **#1 AND #2** |
|  |  |
| ***CINAHL Search Strategy*** | |
| #1 | TX CAA OR Cerebral Amyloid Angiopathy OR Cerebral Amyloid Angiopathies OR Congophilic Angiopathy OR Congophilic Angiopathies |
| #2 | TX ICH OR Intracerebral Hemorrhage OR Intracerebral Hemorrhages OR Cerebral Hemorrhage OR Cerebral Hemorrhages OR Cerebrum Hemorrhage OR Cerebrum Hemorrhages OR Cerebral Parenchymal Hemorrhage OR Cerebral Parenchymal Hemorrhages OR Cerebral Brain Hemorrhage OR Cerebral Brain Hemorrhages |
| #3 | **#1 AND #2** |

**Supplementary Table 3.** Quality assessment of all Included Study in this Meta-Analysis.

| Study | Selection | | | | Comparability | | Outcome/ Exposure | | | | Scores |
| --- | --- | --- | --- | --- | --- | --- | --- | --- | --- | --- | --- |
|  | Representativeness of the exposed cohort | Selection of the non-exposed cohort | Determination of exposure | Ascertainment of no outcome before the study | Matching the most important factor | Control of confounding factors | Evaluation of outcome | Adequacy of follow up | | Completeness of follow up |  |
| Yanagawa T, 2023 | 1 | 1 | 1 | 0 | 1 | 1 | 1 | 0 | | 0 | 7 |
| Koemans EA, 2023 | 1 | 1 | 1 | 1 | 1 | 1 | 1 | 1 | | 1 | 9 |
| Yang Q, 2022 | 1 | 1 | 1 | 1 | 1 | 1 | 1 | 1 | | 1 | 9 |
| Goeldlin MB, 2022 | 1 | 1 | 1 | 0 | 1 | 1 | 1 | 1 | | 1 | 8 |
| Che R, 2022 | 1 | 1 | 1 | 1 | 1 | 1 | 1 | 1 | | 1 | 9 |
| Xu TQ, 2021 | 1 | 1 | 1 | 1 | 1 | 1 | 1 | 0 | | 1 | 8 |
| Xia M, 2021 | 1 | 1 | 1 | 1 | 1 | 1 | 1 | 1 | | 1 | 9 |
| Tsai HH, 2021 | 1 | 1 | 1 | 0 | 1 | 1 | 1 | 1 | | 0 | 7 |
| Li Q, 2021 | 1 | 1 | 1 | 1 | 1 | 1 | 1 | 1 | | 1 | 9 |
| Raposo N, 2020 | 1 | 1 | 1 | 1 | 1 | 1 | 1 | 1 | | 1 | 9 |
| Cheng X, 2020 | 1 | 1 | 1 | 1 | 1 | 1 | 1 | 1 | | 1 | 9 |
| Charidimou A, 2019 | 1 | 1 | 1 | 1 | 1 | 1 | 1 | 1 | | 1 | 9 |
| Pasi M, 2018 | 1 | 1 | 1 | 1 | 1 | 1 | 1 | 1 | | 1 | 9 |
| Charidimou A, 2017 | 1 | 1 | 1 | 1 | 1 | 1 | 1 | 1 | | 1 | 9 |
| Xia L, 2017 | 1 | 1 | 1 | 1 | 1 | 1 | 1 | 1 | | 1 | 9 |
| Boulouis G, 2017 | 1 | 1 | 1 | 1 | 1 | 1 | 1 | 1 | | 1 | 9 |
| Roongpiboonsopit D, 2016 | 1 | 1 | 1 | 1 | 1 | 1 | 1 | 1 | | 1 | 9 |
| Koo HW, 2016 | 1 | 1 | 1 | 0 | 1 | 1 | 1 | 1 | | 1 | 8 |
| Yeh SJ, 2014 | 1 | 1 | 1 | 1 | 1 | 1 | 1 | 1 | | 0 | 8 |
| van Etten ES, 2014 | 1 | 1 | 1 | 1 | 1 | 1 | 1 | 1 | | 1 | 9 |
| Charidimou A, 2013 | 1 | 1 | 1 | 0 | 1 | 1 | 1 | 1 | | 1 | 8 |
| Biffi A, 2012 | 1 | 1 | 1 | 1 | 1 | 1 | 1 | 1 | | 1 | 9 |
| Domingues-Montanari S, 2011 | 1 | 1 | 1 | 1 | 1 | 1 | 1 | 1 | | 1 | 9 |
| Biffi A, 2010 | 1 | 1 | 1 | 1 | 1 | 0 | 1 | 1 | | 1 | 8 |
| Petridis AK, 2008 | 1 | 1 | 1 | 0 | 1 | 1 | 1 | 1 | | 1 | 8 |
| Lzumihara A, 2005 | 1 | 1 | 1 | 0 | 1 | 1 | 1 | 0 | | 1 | 7 |
| Greenberg SM, 2004 | 1 | 1 | 1 | 1 | 1 | 1 | 1 | 1 | | 1 | 9 |
| O’Donnell HC, 2000 | 1 | 1 | 1 | 1 | 1 | 1 | 1 | 1 | | 1 | 9 |
|  | Adequacy of case definitions | Representativeness of the Cases | Selection of Controls | Definition of Controls | Comparability of Cases and Controls on the Basis of the Design or Analysis | | Ascertainment of Exposure | | Non-Response Rate | |  |
| Garg A, 2022 | 1 | 1 | 1 | 1 | 1 | | 1 | | 1 | | 9 |
| Pinho J, 2021 | 1 | 1 | 1 | 1 | 1 | | 1 | | 1 | | 9 |

**Supplementary Table 4.** Results of Meta-Analysis of Risk Factors Associated with Recurrence of CAA-ICH.

| Risk factors | Number of Included studies | Number of patients | Heterogeneity | | Analysis model | Pooled effects | |
| --- | --- | --- | --- | --- | --- | --- | --- |
|  |  |  | I^2^ (%) | P value |  | OR (95%CI) | P value |
| Age | 11 | 9436 | 70.4 | <0.001 | random | 1.02(0.99, 1.05) | 0.19 |
| Gender | 7 | 8698 | 0 | 0.99 | fixed | 0.95(0.77, 1.17) | 0.62 |
| Hypertension | 7 | 8498 | 29.9 | 0.20 | fixed | 0.81(0.61, 1.08) | 0.15 |
| Diabetes | 6 | 8458 | 2.9 | 0.40 | fixed | 0.87(0.64, 1.19) | 0.39 |
| Dyslipidemia | 4 | 8319 | 0 | 0.80 | fixed | 1.02(0.8, 1.30) | 0.88 |
| Previous ICH | 8 | 1626 | 36.8 | 0.14 | fixed | 2.03(1.5, 2.75) | <0.001 |
| Anticoagulation | 3 | 477 | 74.2 | 0.02 | random | 1.72(0.16, 18.13) | 0.651 |
| Antiplatelet therapy | 4 | 581 | 69.7 | 0.02 | random | 1.66(0.66, 4.15) | 0.28 |
| Baseline ICH volume | 4 | 442 | 0 | 0.51 | fixed | 1.01(1, 1.02) | 0.004 |
| IVH presence | 2 | 326 | 0 | 0.36 | fixed | 1.35(0.49, 3.78) | 0.56 |
| cSAH presence | 3 | 721 | 0 | 0.44 | fixed | 3.05(1.86, 4.99) | <0.001 |
| Lobar CMBs count | 5 | 585 | 0 | 0.52 | fixed | 1(0.99, 1) | 0.57 |
| Lobar CMBs>5 | 4 | 477 | 75.3 | 0.007 | random | 1.61(0.54, 4.81) | 0.39 |
| cSS presence | 5 | 898 | 0 | 0.94 | fixed | 2.04(1.46, 2.83) | <0.001 |
| Disseminated cSS | 6 | 962 | 16 | 0.31 | fixed | 3,21(2.25, 4.58) | <0.001 |
| Focal cSS | 3 | 373 | 0 | 0.98 | fixed | 1.46(0.78, 2.74) | 0.24 |
| CSO-PVS | 4 | 430 | 0 | 0.45 | fixed | 1.67(1.14, 2.45) | 0.009 |
| WMH volume | 3 | 537 | 0 | 0.81 | fixed | 1(1, 1.01) | 0.517 |
| Total MRI buden of SVD | 2 | 297 | 77.1 | 0.04 | random | 1.63(0.9, 2.9) | 0.104 |

OR: odds ratio.

## Supplementary Figure


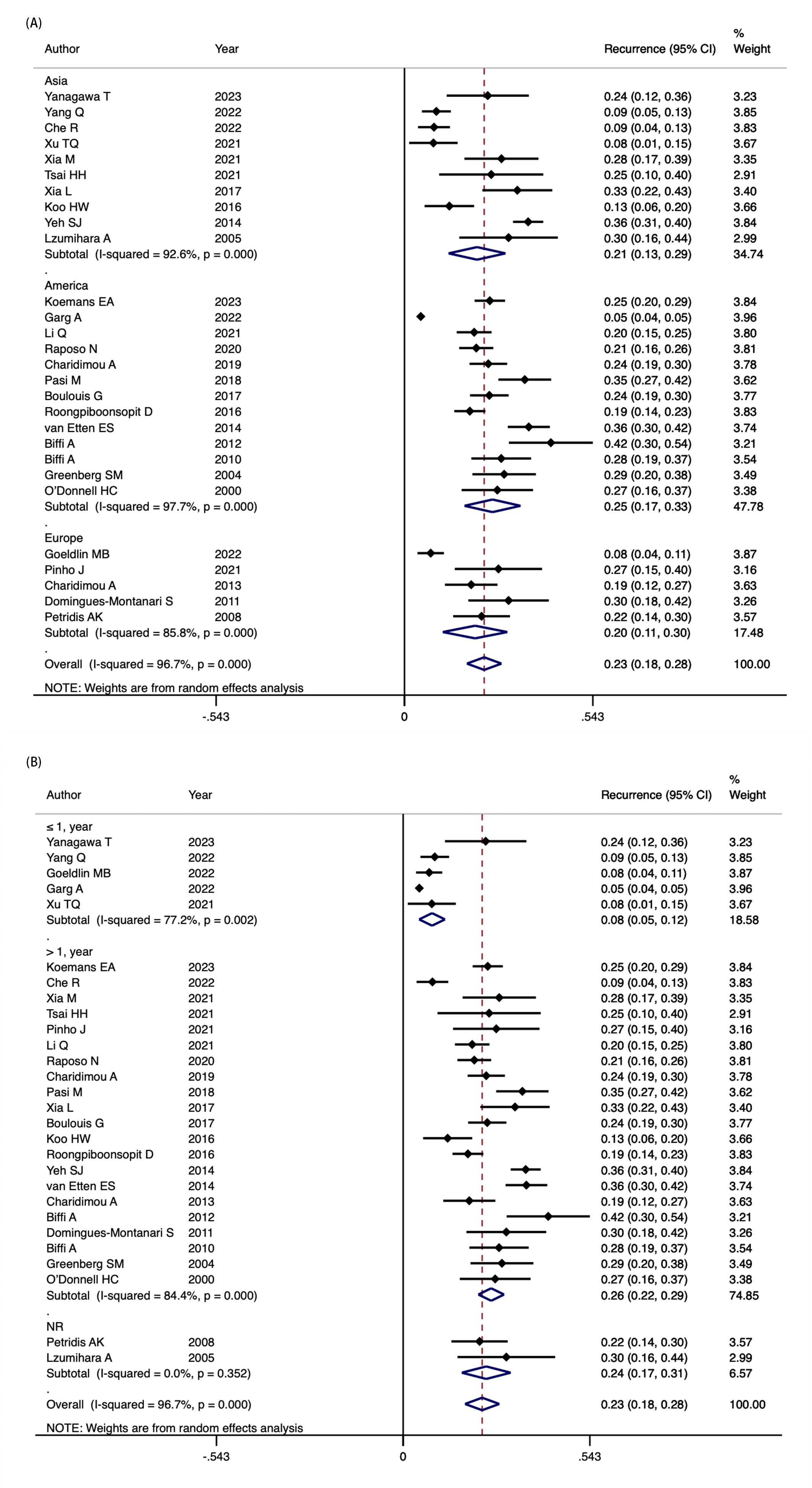


**Supplementary Figure 1.** Results of subgroup analysis of recurrence rate (A) Subgroup analysis based on region; (B) Subgroup analysis based on follow-up duration.
